# Dexmedetomidine vs. midazolam-fentanyl for intraoperative analgesia and sedation: a systematic review and meta analysis

**DOI:** 10.3389/fpain.2026.1664460

**Published:** 2026-03-27

**Authors:** Hashim Talib Hashim, Mostafa A. Khalifa, Aya Ahmed Shimal, Marafi Jammaa Ahmed, Mohamed H. Elbadawi, Khadeeja Ali Hamzah, Abdulhadi M. A. Mahgoub, Alaa R. AL-Ihribat, Salem Waleed Salem Mohamed, Fathima Raahima Riyas Mohamed, Elian Khalafalla, Amna Kamil, Anzah Imtiaz Wagga, Ahmed Mohamed Shahin, Abdelrhman H. Mohammed

**Affiliations:** 1 College of Medicine, University of Warith Al-Anbiyaa, Karbala, Iraq; 2 Faculty of Medicine, Cairo University, Cairo, Egypt; 3 College of Medicine, University of Baghdad, Baghdad, Iraq; 4 Faculty of Medicine, Bahri University, Khartoum, Sudan; 5 Faculty of Medicine, University of Khartoum, Khartoum, Sudan; 6 Baghdad University AIkindy College of Medicine, Baghdad, Iraq; 7 Faculty of Medicine, University of Gezira, Madani, Gezira, Sudan; 8 College of Medicine and Health Sciences, Polytechnic University, Hebron, Palestine; 9 Faculty of Medicine, Alexandria University, Alexandria, Egypt; 10 College of Medicine, Alfaisal University, Riyadh, Saudi Arabia; 11 Jinnah Sindh Medical University, Karachi, Pakistan; 12 Faculty of Medicine, Menoufia University, Menoufia, Egypt; 13 College of Medicine, Luxor University, Luxor, Egypt

**Keywords:** analgesia, dexmedetomidine, midazolam, midazolam-fentanyl, sedation

## Abstract

**Background:**

Ongoing research aims to identify the most effective sedative for procedural sedation. This meta-analysis compares dexmedetomidine with midazolam-fentanyl in surgical patients.

**Methods:**

We conducted a search through MEDLINE, EMBASE and CENTERAL to find randomized controlled trials (RCTs) comparing dexmedetomidine and midazolam-fentanyl. Data on participant characteristics, intervention details, and outcomes were extracted, focusing on intraoperative hemodynamic parameters, respiratory safety, and adverse events. Sedative efficacy and recovery profiles were also evaluated and assessed descriptively across included studies. Data were analyzed using RevMan 5.4, applying random effects models for significant heterogeneity (I^2^ > 50%). Studies with fewer than three data points per group were excluded, and sensitivity analyses were performed.

**Results:**

We include 4 RCTs with 259 patients. Dexmedetomidine significantly lowered mean arterial pressure (MAP) by −6.42 mmHg (95% CI: −8.24 to −2.21, *p* < 0.001). Initially, heart rate differences were not significant, but after excluding an outlier, dexmedetomidine reduced heart rates by 6.71 bpm (95% CI: −10.74 to −2.68, *p* = 0.001). Respiratory rate and oxygen saturation showed no significant differences between groups, and adverse events were comparable.

**Conclusion:**

Dexmedetomidine offers superior blood pressure control compared to midazolam-fentanyl, with similar safety profiles for respiratory parameters. Further research is needed to assess long-term outcomes such as postoperative delirium and residual sedation.

## 1 Introduction

Patients admitted to the ICU are usually in need of invasive and uncomfortable interventions such as mechanical ventilation. To reduce anxiety, increase tolerance, ease patient comfort and safety and improve outcomes of such interventions, sedation is common practice (1). Intraoperative sedation is essential for maintaining patient comfort, optimizing surgical conditions, and ensuring hemodynamic stability during surgery (2). Many drugs are used to reduce anxiety and induce sedation, such as benzodiazepines, barbiturates, phenothiazines, opioids, and antihistamines, but these agents can also prolong mechanical ventilation and ICU stay (3).

Traditionally, sedative agents administered in the ICU are *γ*-aminobutyric receptor agonists (GABA) which include the benzodiazepines such as Midazolam (1). Although midazolam is believed to have few hemodynamic side effects, it is one of the most frequently used sedatives for procedural sedation (2). A combination of midazolam, a benzodiazepine, and fentanyl, an opioid, has been widely used due to their synergistic effects in achieving the desired depth of sedation and analgesia. Midazolam is known for its rapid onset and short-lasting effect and sedative-hypnotic properties, while fentanyl provides potent analgesia. Yet, opioids are well known to produce tolerance, dependence, and a number of unwanted side effects such as respiratory depression, prolonged sedation, apnea, loss of airway reflexes and challenges in withdrawal (4).

Dexmedetomidine (an alpha2-adrenergic agonist) is a selective, specific, and highly potent new drug, which can also be used for procedural sedation. It has sedative, hypnotic and anxiolytic properties and is known for its analgesic potential owing to a decrease in sympathetic tone (2). It is a highly selective alpha-2 receptor agonist target at the locus coeruleus, providing an alternative to a GABA agonist for ICU sedation (5). It has less of an impact on arousability and patient interaction (6). Dexmedetomidine is also noted for its role in enhancing patient comfort, enabling faster recovery, and facilitating earlier extubation (7). However, the United States Federal Drug Administration has advised that it is only used for short-term sedation (<24 h) because of its dose-dependent cardiovascular effects, such as reductions in heart rate, cardiac output, arterial and pulmonary arterial pressures and complications of respiratory failure, such as acute respiratory distress syndrome (6). Dexmedetomidine can also lower the incidence of postoperative delirium, rather than the more common sedatives (propofol or midazolam), a significant and somewhat common complication in surgical patients that is defined by a transient mental state that includes hallucinations, confusion, anxiety, and incomprehensible speech (8). A recent meta-analysis, however, produced conflicting findings on the impact of dexmedetomidine on duration of mechanical ventilation and ICU stay (9). In actual clinical practice, midazolam is still commonly used, even though recent international guidelines provided a conditional recommendation favoring the use of dexmedetomidine over benzodiazepines in adults on mechanical ventilation, particularly during the COVID-19 pandemic when medication shortages occurred (10).

Many studies have compared aspects of the safety and efficacy of midazolam and dexmedetomidine, but the results have not yet been systematically reviewed. The objective of this systematic review and meta-analysis is to comprehensively compare the efficacy, safety, and patient outcomes of dexmedetomidine vs. midazolam-fentanyl for sedation and analgesia when used as monosedatives. We also assessed whether sedation with dexmedetomidine is safer and more efficacious than standard sedation with a midazolam-fentanyl regimen in maintaining the target sedation level in long-stay ICU patients. By consolidating findings from multiple studies, this review will provide evidence to guide intraoperative sedation practices and may help inform updates to surgical anesthesia protocols.

## 2 Methods

This systematic review adhered to the PRISMA-2020 guidelines (Preferred Reporting Items for Systematic Reviews and Meta-Analyses), maintaining a consistent and structured approach through each phase of the research process, from the literature search to data synthesis. The review's aims and scope were defined using the PICO framework (Participants, Interventions, Comparisons, and Outcomes), as outlined in Table 1.

**Table 1:** PICO framework.

PICO	Description
P (participants)	Patients undergoing surgery require intraoperative sedation and analgesia.
I (intervention)	Dexmedetomidine administration during surgery.
C (comparisons)	Midazolam combined with fentanyl during surgery
O (outcomes)	Hemodynamic parameters, respiratory safety, adverse events, and immediate recovery characteristics.

A comprehensive search strategy was developed on 3 of March 2025 to identify studies examining the effects of dexmedetomidine vs. midazolam-fentanyl on postoperative hemodynamic changes. The search spanned major databases: MEDLINE, EMBASE and CENTERAL for their extensive coverage of medical and clinical literature. To ensure retrieval accuracy, the search query incorporated a combination of keywords and Medical Subject Headings (MeSH) terms, using phrases such as (Dexmedetomidine OR Dexmedetomidine Hydrochloride OR MPV-1440 OR Igalmi OR Percedex OR Sedadex OR Sileo OR Cepedex OR Dexdor OR Dexdomitor OR DEX) AND (Midazolam OR Dormicum OR Ro 21-3981 OR Midozolam Hydrochloride OR Midozolam Maleate OR MDZ) AND (Sedation OR Analgesia OR Pain Control OR Anesthesia OR Intensive Care). Boolean operators (AND/OR) were applied to structure the syntax effectively, ensuring the identification of studies that specifically evaluated the impact of dexmedetomidine and midazolam on postoperative hemodynamic parameters, sedation quality, and clinical outcomes. The search spanned titles, abstracts, and keywords. All citations were imported into the Rayyan software for processing, including duplicate removal and initial screening.

Inclusion and exclusion criteria were meticulously defined to ensure that only scientifically rigorous studies were considered. Eligible studies had to be peer-reviewed original research articles published in English, focusing on adult intraoperative patients patients who received either dexmedetomidine or midazolam for sedation. Only studies that directly compared dexmedetomidine with midazolam (with or without fentanyl) within the same study population were included. The primary outcomes assessed included hemodynamic parameters such as blood pressure, heart rate, and cardiac output, along with sedation efficacy and postoperative recovery. Eligible study design are only randomized control trial. Studies were excluded if they were non-peer-reviewed sources (such as editorials, opinion pieces, conference abstracts, or indexes), focused on sedatives other than dexmedetomidine or midazolam, or did not report relevant hemodynamic outcomes. Studies reporting outcomes for only one sedative agent were excluded to avoid indirect comparisons and excessive clinical heterogeneity. Articles written in languages other than English were also excluded from the review.

The screening process involved two reviewers [A.M., K.A.]. Discrepancies among reviewers were addressed through discussion and the senior author's advice.

We followed a systematic approach, beginning with title screening, followed by abstract screening, and culminating in full-text review. Each article's title and abstract were initially assessed against predefined inclusion and exclusion criteria. The subsequent full-text evaluation ensured that studies met all eligibility requirements and provided sufficient data on postoperative hemodynamic effects. This structured three-phase screening process facilitated the selection of studies that directly addressed the research objectives, ensuring the inclusion of high-quality evidence.

Data extraction was performed using a structured Microsoft Excel form to systematically capture relevant information. Extracted data included study identification details (first author, publication year, study title, and design), participant characteristics (sample size, age, gender, surgical procedure, and postoperative sedation requirements), and intervention details (drug administered, dosage, administration route, and duration). Outcome variables focused on hemodynamic parameters (blood pressure, heart rate, cardiac output), and adverse events. Sedative efficacy and recovery-related outcomes were inconsistently reported across studies and, therefore, were summarized narratively rather than pooled quantitatively.

To assess the risk of bias in the included studies, the Cochrane bias assessment tool was applied based on study design, which is randomized controlled trials of 4 RCTs (11), to ensure a robust and standardized evaluation. We involved two reviewers [SW and AK]. Discrepancies among reviewers were addressed through discussion and the senior author's advice.

Meta-analysis was conducted using RevMan software V5.4. We assessed the heterogeneity using the I^2^ statistic. The I^2^ statistic quantifies the proportion of total variability in effect estimates that is attributable to between-study heterogeneity rather than chance. Then, a random-effects model was applied when significant heterogeneity was present; otherwise, a fixed-effects model was applied. An I^2^ value below 50% indicates the absence of heterogeneity. An I^2^ value greater than 50% was considered indicative of substantial heterogeneity, reflecting meaningful clinical or methodological differences between included studies.

Mean difference was used to present the effect sizes of the outcomes. Outcomes which were included in fewer than 3 studies (for each group of comparators) weren't included in the analysis. In all outcomes, we analysed the parameters at the end time point of follow-up. Assessment of heterogeneity is essential in meta-analysis because high between-study variability may influence the reliability and interpretation of pooled estimates. Heart rate and mean arterial pressure were analyzed as changes from baseline to the final reported intraoperative time point, as defined by the original studies. Sensitivity analysis was also performed to determine the causes of heterogeneity. Standard deviations (SD) were obtained from a comparable study with a similar type of surgery, gender distribution, and long follow-up duration (12). These values were used to impute missing SDs in another study that did not report them (13).

## 3 Results

### 3.1 Search results and study selection

**Figure 1:**
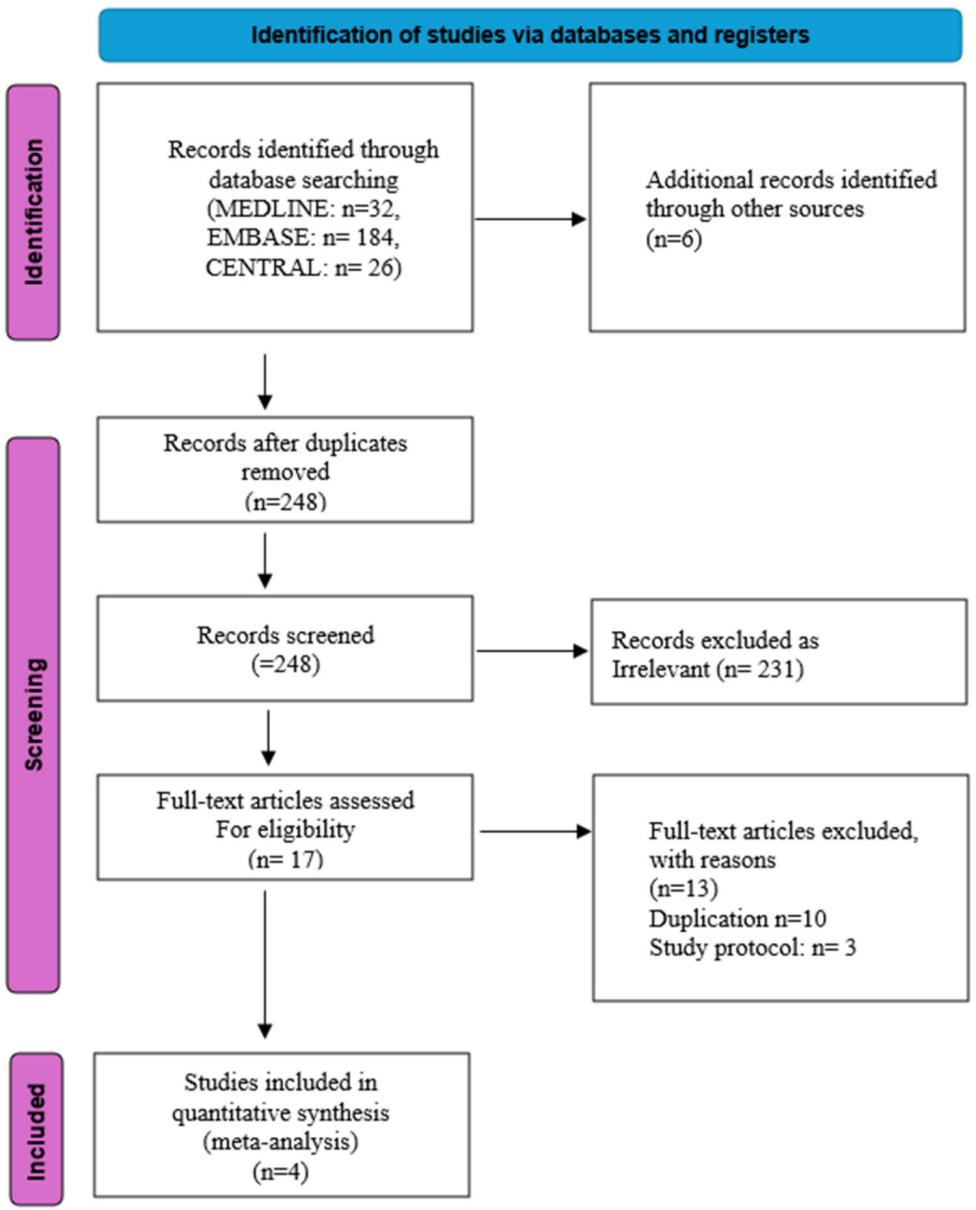
Preferred reporting items for systematic reviews and meta-analyses (PRISMA) flow diagram.

The literature search identified 248 records after removal of duplicates, including records retrieved from electronic databases and additional sources. All records were screened based on titles and abstracts, resulting in the exclusion of 231 articles that did not meet the inclusion criteria. Seventeen full-text articles were assessed for eligibility, of which 13 were excluded for predefined reasons, including duplication (*n* = 10) and study protocols (*n* = 3). Ultimately, four randomized controlled trials met all eligibility criteria and were included in the quantitative synthesis. PRISMA flowchart [Figure 1] illustrates the selection procedure.

### 3.2 Baseline characteristics of the included studies

The number of included studies was 4 studies. All of the included studies were randomized controlled clinical trials which targeted the use of dexmedetomidine vs. midazolam among surgery patients. All studies targeted adult patients. The total sample size was 259, with a mean age of 33.36 ± 18.3 years old. The mean duration of surgery was 63.5 ± 50 min. The mean weight across studies was 55.9 ± 21.0 kg. The monitoring period ranged from 6 min after anesthesia induction to 180 min (Table 2).

**Table 2:** Characteristics of included studies.

Study	Sample size	Age (mean and SD) (years)	Duration of surgery (mean and SD) (minutes)	Females (*N*, %)	Weight (mean and SD) (kg)	Type of surgery	Duration of intraoperative monitoring after induction (minutes)
Zeyneloglu, (1)	49	47.8 ± 12	39.71 ± 7.9	23 (46.94%)	76.6 ± 14	Outpatient extracorporeal shock wave lithotripsy (ESWL)	50
Jaakola, (2)	20	45.5 ± 4.7	10.98 ± 11.3	20 (100%)	67 ± 10	Elective abdominal hysterectomy	6
Parikh, (3)	90	29.3 ± 11.4	79.75 ± 11	48 (53.33%)	53.6 ± 10.7	Tympanoplasty	90
Oriby, (4)	60	42.9 ± 15.3	14.13 ± 6.6	32 (53.33%)	64.7 ± 5.7	Microlaryngoscopy	10

### 3.3 Risk of bias assessment

**Figure 2:**
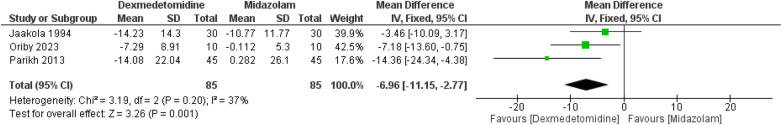
Fixed effect model for the change in heart rate after removal of the outlier.

**Figure 3:**
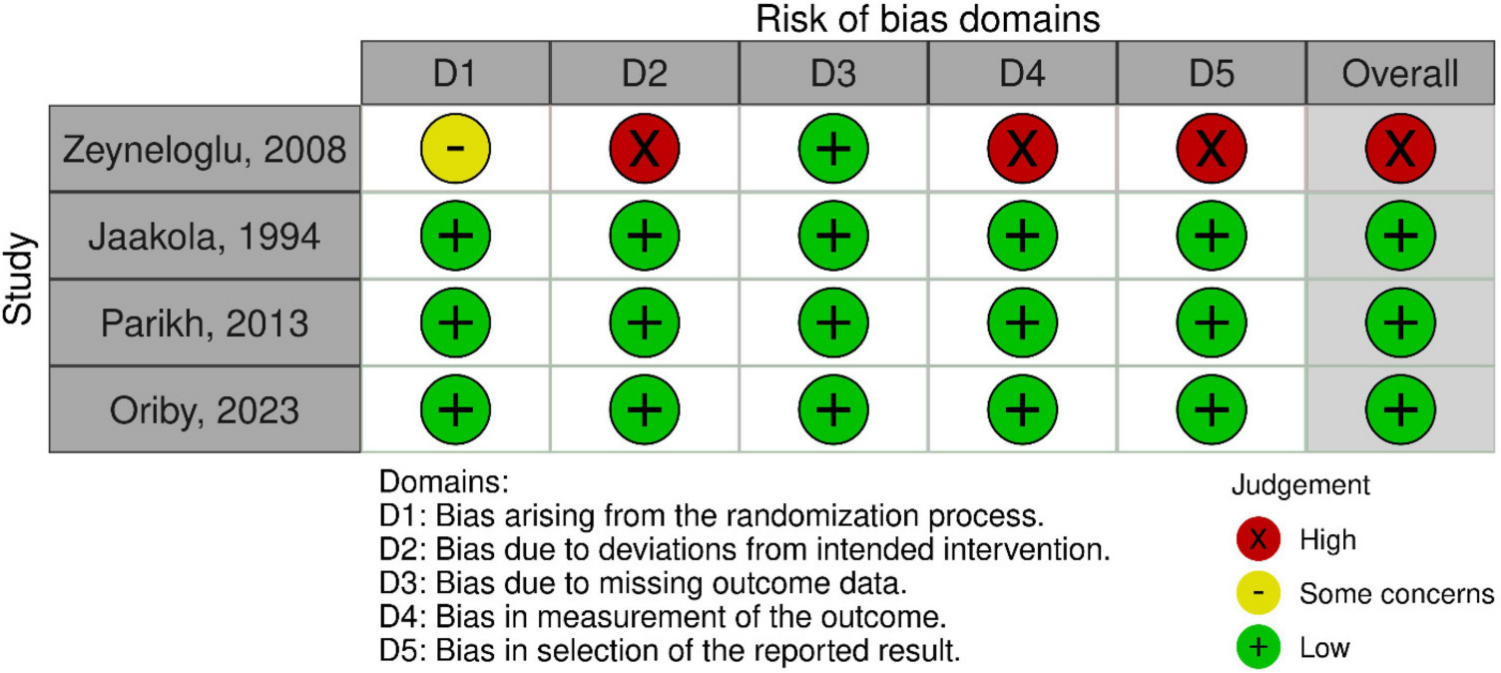
Risk of bias assessment of the included studies.

**Figure 4:**
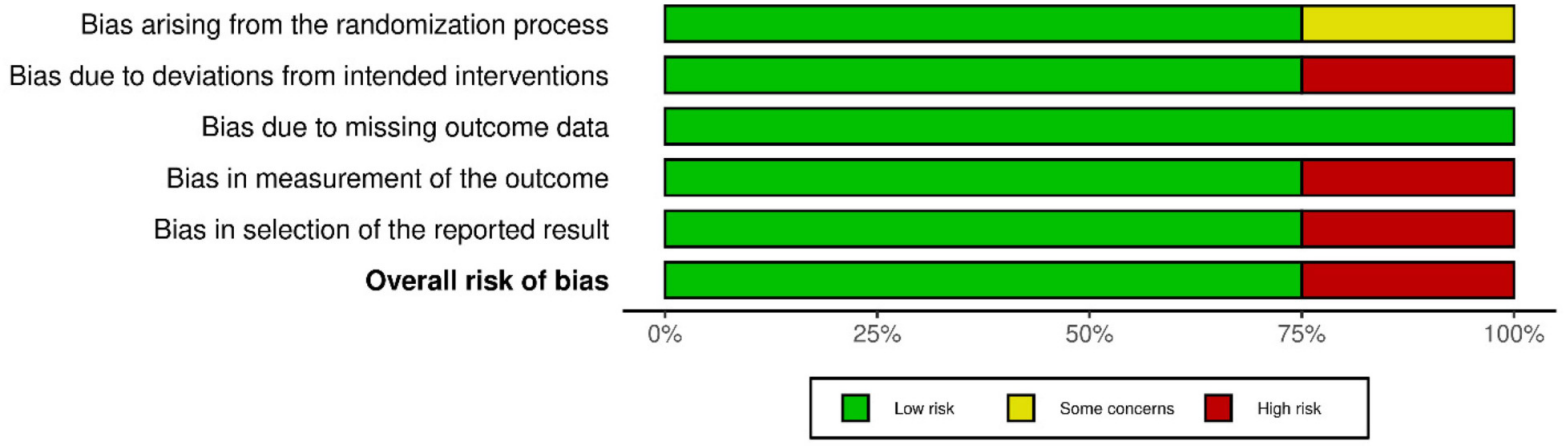
Summary of the risk of bias assessment.

According to the ROB-2 assessment (Figure 2), one study was judged to have a high risk of biasone study was judged to have a high risk of bias (14), while three studies were rated as low risk of bias (12, 15, 16), mainly due to concerns in deviations from intended interventions, measurement of outcomes, and selective reporting as presented in Figures 3,4.

### 3.4 Outcomes

#### 3.4.1 Change in heart rate

**Figure 5:**
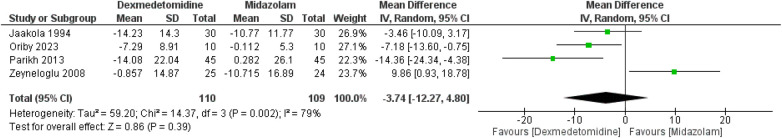
Random effect model for the change in heart rate.

Random effect model was performed on 4 studies with 110 patients in the Dexmedetomidine group and 109 in the midazolam group, the pooled estimate of change in heart rate was −3.74 beats per minute (C.I = −12.27, 4.80), but the difference between Dexmedetomidine and Midazolam wasn't significant (*p*-value = 0.39). These findings can't be generalized due to the use of a random effect model implied by the high significance of heterogeneity (I^2^ = 79%, *p*-value=0.002) (Figure 5). Sensitivity analysis was performed by removing one study at a time. Removal of each study didn't eliminate heterogeneity except when removal of Zeyneloglu, 2008 study (14). After removal of the outlier (14), the Dexmedetomidine group showed significantly more reduction in heart rate by 6.96 beats per minute than the midazolam group in the fixed effects model (C.I = −11.15, −2.77) (*p*-value = 0.001) (Figure 2).

#### 3.4.2 Change in mean arterial pressure

**Figure 6:**
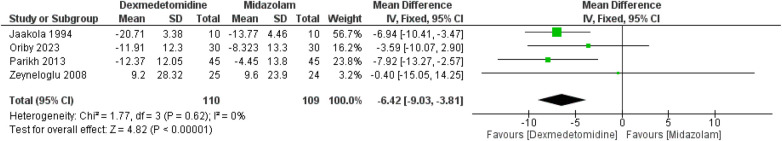
Fixed effects model for the change in mean arterial pressure.

Fixed effects model was performed on 4 studies with 110 patients in the Dexmedtomidine group and 109 in the midozolam group, the pooled estimate for the change in mean arterial pressure was −6.42 mmHg (C.I = −9.03, 3.81) (*p*-value <0.001), indicating significantly more reduction in mean arterial pressure in the Dexmedetomidine group than in the midazolam group (Figure 6).

#### 3.4.3 Respiratory rate

Only two studies reported the respiratory rate (12, 14). In both studies, there was no significant difference between the Dexmedetomidine group and the midazolam group at the end of the follow-up periods.

#### 3.4.4 Oxygen saturation

Only two studies reported the oxygen saturation (14, 15). Both of these studies reported no significant difference in oxygen saturation at the end of the study. Jaakola, 1994 study reported almost normal saturation across the period of the study, with the smallest values being 96% and 95% in the Dexmedetomidine group and the midazolam group, respectively. While in Zeyneloglu, 2008 reporter abnormal findings, with the smallest values to be 92.7% and 92.3% in the Dexmedetomidine group and the midazolam group, respectively (14).

#### 3.4.5 Adverse events

Multiple adverse events have been reported across the studies, which were: nausea, vomiting, hypotension, hypertension, hypoxemia, bradycardia and agitation. Regarding Zeyneloglu, 2008 study, incidences of nausea and vomiting weren't significantly different between Dexmedetomidine and Midazolam groups (14). Hypotension incidences weren't found to be significantly different between the groups in both Zeyneloglu, 2008 and Oriby, 2023 studies (14, 16). Bradycardia incidence was zero in Zeyneloglu, 2008 study (14), but reached 10 cases (16.7%) in Oriby, 2023 study (16), with no significant difference between groups. Hypoxemia and hypertension were only mentioned in one study (Zeyneloglu, 2008), with no significant difference between Dexmedetomidine and midazolam groups (14).

## 4 Discussion

Advancements in sedative agents have reshaped perioperative care, optimizing both pain management and patient recovery outcomes. Dexmedetomidine, an alpha-2 adrenergic agonist, has gained attention for its sedative properties while preserving respiratory function (17). Midazolam, a benzodiazepine, is often combined with fentanyl to provide sedation and analgesia during surgical procedures (18). Despite their widespread use, the comparative efficacy and safety of dexmedetomidine vs. midazolam-fentanyl remain a topic of debate. This meta-analysis aimed to provide a comprehensive comparison between these sedative strategies in patients undergoing procedures requiring sedation and analgesia.

Our meta-analysis of four randomized controlled trials (RCTs), encompassing 259 patients, revealed key differences between dexmedetomidine and midazolam-fentanyl in hemodynamic parameters, particularly mean arterial pressure (MAP) and heart rate (HR). While the pooled estimate for change in HR did not initially show statistical significance, sensitivity analysis revealed that after removing the outlier study (Zeyneloglu, 2008) (14), dexmedetomidine significantly reduced HR compared to midazolam. Additionally, dexmedetomidine led to a significantly greater reduction in MAP. These findings align with previous research by Wen et al. (10) demonstrating dexmedetomidine's sympatholytic effects, which contribute to hemodynamic stability and reduced cardiovascular stress. However, differences in surgical types and anesthetic techniques among the included studies limit direct comparison. However, its impact on bradycardia remains controversial, as some studies have reported an increased incidence of bradycardia with dexmedetomidine use (19, 20).

In terms of respiratory safety, our findings suggest no significant differences in respiratory rate or oxygen saturation between the two groups, consistent with prior meta-analysis by Lin et al. (21). Notably, dexmedetomidine's ability to provide sedation without significant respiratory depression is a crucial advantage in procedural sedation settings. Previous literature has emphasized the respiratory risks associated with benzodiazepines and opioids, particularly in vulnerable populations (22, 23).

Regarding adverse events, our analysis highlighted variability across studies and did not find significant differences. Similarly, hypotension and bradycardia occurred in both groups without a clear pattern of predominance. This variability may stem from differences in dosing protocols, patient characteristics, and monitoring durations across studies.

The heterogeneity observed in our analysis was particularly notable in HR outcomes, necessitating the use of a random-effects model. Potential sources of heterogeneity include variations in patient demographics, surgical procedures, and anesthetic protocols. For instance, the duration of follow-up varied significantly across studies, potentially affecting outcome assessment.

Nevertheless, based on the current evidence, dexmedetomidine provides superior hemodynamic stability compared with midazolam-fentanyl while maintaining a comparable safety profile for respiratory outcomes. These advantages make dexmedetomidine a valuable option for intraoperative sedation, particularly in patients at risk of cardiovascular stress or opioid-related respiratory depression.

## 5 Limitations

Several limitations must be acknowledged. First, the small number of included studies (*n* = 4) and the relatively modest sample size (259 patients) limit the generalizability of our findings. Second, variations in study designs, including differences in sedative dosing and monitoring protocols, introduce potential bias. Third, the lack of long-term follow-up data prevents assessment of prolonged effects on recovery and patient satisfaction. Finally, publication bias cannot be excluded, as negative findings may be underreported in the literature.

Another key limitation is that our analysis did not capture clinically important outcomes such as postoperative delirium, residual sedation, and difficulty with weaning from sedation. These are highly relevant for clinical practice and should be prioritized in future research.

### 5.1 Implications for future research

Further research is warranted to better delineate the optimal sedation strategy in different procedural settings. Large-scale, multicenter RCTs with standardized protocols are needed to confirm our findings and explore additional outcomes such as recovery time, patient satisfaction, and long-term hemodynamic effects. Additionally, studies evaluating the cost-effectiveness of dexmedetomidine vs. midazolam-fentanyl could provide valuable insights for clinical decision-making.

## 6 Conclusion

This systematic review and meta-analysis demonstrate that dexmedetomidine provides superior hemodynamic stability compared with midazolam-fentanyl, while maintaining a comparable respiratory safety profile. These findings support the use of dexmedetomidine as a preferred sedative strategy in surgical patients, particularly those at higher risk of cardiovascular fluctuations or opioid-related respiratory compromise. Clinicians should consider incorporating dexmedetomidine more broadly into perioperative protocols to enhance patient safety and optimize surgical outcomes.

## Data Availability

The original contributions presented in the study are included in the article/Supplementary Material, further inquiries can be directed to the corresponding author/s.
